# Keeping Antibiotics at Home Promotes Self-Medication with Antibiotics among Chinese University Students

**DOI:** 10.3390/ijerph15040687

**Published:** 2018-04-05

**Authors:** Xiaomin Wang, Leesa Lin, Ziming Xuan, Lu Li, Xudong Zhou

**Affiliations:** 1 Zhejiang University Institute of Social Medicine, 866 Yuhangtang Road, Hangzhou 310058, Zhejiang, China; ellen_wang@zju.edu.cn; 2 Department of Social and Behavioral Sciences, Harvard T.H. Chan School of Public Health, 677 Huntington Avenue, Boston, MA 02115, USA; llin@hsph.harvard.edu; 3 Center for Community-Based Research, Dana-Farber Cancer Institute, 450 Brookline Avenue, Boston, MA 02215, USA; 4 Division of Policy Translation and Leadership Development, Harvard T.H. Chan School of Public Health, 677 Huntington Avenue, Boston, MA 02115, USA; 5 Department of Community Health Sciences, Boston University School of Public Health, 801 Massachusetts Ave, Boston, MA 02118, USA; zxuan@bu.edu

**Keywords:** self-medication, prophylaxis, keep antibiotics, antimicrobial resistance

## Abstract

Background: Inappropriate use of antibiotics has caused antimicrobial resistance, presenting a major health challenge to the world population. Self-medication with antibiotics (SMA) is currently at an alarming level in China. Objectives: To determine the sources of antibiotics leftover at home, the risk factors of keeping antibiotics at home, and the associations between keeping antibiotics at home and SMA among Chinese university students. Methods: Six provinces were purposely selected from six regions of China, and one multi-disciplinary university was selected from each chosen province. A total of 11,192 university students were selected using cluster random sampling from the selected universities. Logistic regression was conducted to examine the relationship between keeping antibiotics at home and SMA. Results: Out of the 11,192 students surveyed, 7057 (63.1%) reported keeping antibiotics at home at the time they were surveyed. Of those who kept antibiotics in their home, 1965 (27.8%) reported that these antibiotics were left over from a previous prescription by a doctor and 4893 (69.3%), purchased antibiotics over the counter. Additionally, 29.6% (507/1711) of students self-medicated with antibiotics when ill during the month before the survey. Students keeping antibiotics at home were five times (95% CI 3.53, 7.05) more likely to engage in SMA when ill and 2.6 times (95% CI 2.34, 2.89) more likely to self-medicating with antibiotics for prophylaxis than the other students. Female students, those with a family background of higher education, and those who had a parent working in the medical field had a significantly higher chance of keeping antibiotics at home. Conclusions: The high prevalence of keeping antibiotics at home and SMA among young adults is a serious concern. Professional regulations and population-tailored health education are needed.

## 1. Introduction

Antimicrobial resistance (AMR) is a major health challenge in the world [1]. Antibiotics, one of the most important modern medical achievements, are becoming less effective due to inappropriate use. Bacteria are gradually becoming resistant to antibiotics and resistant bacteria have spread worldwide, indicating that we may be at the dawn of a post-antibiotics era [2,3,4]. Addressing the problem of AMR has become a global effort in recent years. In 2016, the General Assembly of the United Nation held a high-level meeting on AMR and passed a political declaration of global cooperation in fighting AMR [5]. AMR was also a high priority issue in the agenda of the Group of Seven (G7) and the Group of Twenty (G20) [6]. 

Inappropriate use of antibiotics for self-limiting diseases is a common and important cause of AMR globally [7,8]. Individual self-medication with antibiotics (SMA) is one of the riskiest antibiotic use behaviors in both developed and developing countries [9,10,11]. Self-medication is recommended by the World Health Organization (WHO) to treat self-recognized disorders or symptoms, or to treat chronic or recurring diseases or symptoms, using drugs prescribed by doctors [12]. Although self-medication in general has been reported to have benefits [13], self-medication specifically with antibiotics is a dangerous practice due to incorrect self-diagnosis, wrong choice of antibiotics, insufficient dosage, allergic reactions, and—more importantly—the possibility of increased resistance to human pathogens [10,14,15,16]. The global prevalence of SMA varies widely, with an average rate of 19.1% in European countries and as high as 91.4% in Nigeria [17,18]. SMA in China is alarmingly common, with a rate of 59.4% in children over the past 12 months and 40.2% among university students over the past 6 months [19,20]. 

Antibiotics used for self-medication come from three sources: antibiotics leftover at home, antibiotics directly purchased over the counter, and antibiotics shared by friends or relatives [21,22]. The two main sources of antibiotics leftover at home are being left over either from a previous prescription by a doctor or from a previous over-the-counter purchase [23,24]. Among most developed countries, antibiotics leftover from a previous prescription by a doctor account for the overwhelming majority of such drugs, and few are left over from previous over-the-counter purchases due to strict regulations [24]. According to a global survey, the rate of leftover antibiotics from a previous prescription in developed countries ranged from 13.5% (in the Netherlands) to 67.5% (in Italy) [25]. These leftover medications are often the result of over-dispensing and non-compliance. In developing countries, both leftover medications from previous treatments and over-the-counter purchases play important roles [26,27], due to the absence of either prescription-only policies or the enforcement of such policies [9,28]. For example, although it has been illegal for pharmacies to sell any types of antibiotics without a prescription since 2004 in China, the antibiotics can be easily purchased from the pharmacies without a prescription. However, little research to date has studied the source of antibiotics left over from previous over-the-counter purchases or the proportion of the two aforementioned sources in developing countries [23]. Understanding the exact proportion of sources of leftover antibiotics will help policy makers address the main problems of inappropriate antibiotic use and develop targeted interventions that focus on the most cost-effective ways to reduce and prevent the inappropriate use of antibiotics. 

Keeping antibiotics at home potentiates SMA when ill. This relationship has been noted in previous studies [1,25]. In a study including 19 European countries, the combination of keeping antibiotics at home and intended self-medication was a strong independent predictor of SMA in the previous 12 months [29]. An American study with 400 respondents found that keeping leftover antibiotics was associated with a more than 4 times greater chance of SMA [30]. Likewise, a Mongolian study on 503 caregivers reported a similar finding, with an odds ratio of 6.3 of SMA for themselves and 1.7 for their children [31]. However, the first study mentioned above did not examine the direct association between keeping antibiotics at home and SMA, and both the second and third had a small sample size. Additionally, all of these studies relied on a 6 to 12 month recall period, which is subject to recall bias. Further studies are needed to analyze the direct associations among these factors. Self-medicating with antibiotics for prophylaxis is considered a common behavior in many developing countries [32,33]; however, no studies have focused on the relationship between keeping antibiotics at home and self-medicating with antibiotics for prophylaxis.

With one fifth of the world’s population and high population mobility, China has a disproportionate share of global burden of antibiotic resistance. Both over-prescription of antibiotics by doctors and self-medication with antibiotics by individuals contribute to inappropriate antibiotic use in China. SMA when ill and self-medicating with antibiotics for prophylaxis account for half of inappropriate antibiotic use [34]. University students with various backgrounds are opinion leaders and represent future stakeholders of AMR. By studying them, the present study aims to (1) determine the proportion of the sources of antibiotics leftover at home in China; (2) examine the risk factors of keeping antibiotics at home; and (3) explore the association between keeping antibiotics at home and SMA. 

## 2. Materials and Methods

### 2.1. Participants

Data for this study came from a large cross-sectional survey conducted among university students in China between September and November 2015. Geographically, China has six regions: north, east, northeast, northwest, central-south, and southwest [35]. One province was purposely selected from each region, and one multi-disciplinary university was selected from each chosen province. These include Nankai, Zhejiang, Jilin, Lanzhou, Wuhan, and Guizhou Universities.

### 2.2. Measures

A self-administered questionnaire was utilized, with questions including whether the respondents (i) self-treated with antibiotics without advice from a doctor, nurse, or dentist when ill in the past month; (ii) self-medicating with antibiotics for prophylaxis without advice from a doctor, nurse, or dentist in the past year; (iii) possessed leftover antibiotics (not for current use) in their home at the time of the survey, and if so what the sources of the leftover antibiotics were. Additionally, questions regarding the details of the antibiotic use—such as the names of the medicine and symptoms or diseases—as well as questions regarding the demographic characteristics of the respondents were included. The questionnaire was initially validated through qualitative interviews of 15 university students, followed by a test-retest of 200 participants.

### 2.3. Data Collection

An electronic questionnaire was developed using Wen Juan Xing (Chinese Survey Monkey), an online service that provides professional, user-friendly online questionnaire design and data collection [36]. We used the cluster random sampling method to conduct the survey. A total of 1800 students were selected and targeted to cover disciplines in the sciences, the social sciences/humanities, and in medicine from each university. Classes were randomly selected according to the academic calendar on the main campus and all students in the selected classes were asked to fill out the questionnaire. Three investigators obtained permissions from the class instructors at each university (with none refusing), explained the purpose of the survey, and explained the procedures to fill out the electronic questionnaires to the students before class began. The investigators then disseminated the printed quick response code of the electronic questionnaire and completion of the questionnaire took just a few minutes. The first section of the questionnaire consisted of an information sheet and consent form to be signed by the participants. It was clearly explained that participation was voluntary and over 95% of the students in the selected classes completed the questionnaire. A gratuity of RMB 3 yuan ($0.50 USD) was paid via smartphone to all students who completed the questionnaire. The study was reviewed by the Institutional Review Board of School of Public Health, Zhejiang University (no. ZGL20160922).

### 2.4. Statistical Analysis

Descriptive analyses, expressed as weighted frequencies and percentages, were performed. A logistic regression model (Model 1) was used to determine factors associated with keeping antibiotics at home (Yes/No), SMA when ill (Yes/No), and self-medicating with antibiotics for prophylaxis (Yes/No). In Model 2, we divided keeping antibiotics at home into four groups according to the leftover sources (group 1 = no; group 2 = yes, previously prescribed by doctors; group 3 = yes, previously purchased from pharmacies; group 4 = yes, from other sources). The significance level (type 1 error rate) is set at 0.05. SPSS 22.0 (SPSS Inc., Chicago, IL, USA) was used for the statistical analysis.

## 3. Results

### 3.1. Descriptive Statistics of the Sample

A total of 11,192 respondents completed the questionnaire. A total of 267 questionnaires (2.4%) were discarded because of non-completion of key variables (behavioral variables). Table 1 presents the demographic characteristics of the sample. Female respondents accounted for 50.7%, undergraduates for 79.4%, and medical students for 16.3%. Respondents had an average age of 20.7 years. Students from rural areas accounted for 44% of respondents, 30.5% had a household income of less than 3000 RMB yuan ($461 USD), and 52% had a household income of 3000–10,000 RMB yuan ($462–$1538 USD). It was also observed that 8.7% of the respondents had at least one parent with a medical background. 

Table 1 displays the reported behaviors regarding keeping antibiotics at home at the time of the survey, SMA when ill in the preceding month, and self-medicating with antibiotics for prophylaxis in the preceding year. Out of 11,192 respondents, 7057 (63.1%) had kept antibiotics at home; 1965 (27.8%) reported that these antibiotics were leftovers from a previous prescription by a doctor; 4893 (69.3%), that they were purchased over the counter; and 125 (1.8%) were given to the respondent by others. In the month before the survey, 3337 students reported illness, with the main symptoms including cold (76.1%), sore throat (46.9%), and fever (20.3%). Among respondents reporting such an illness, 1711 (51.3%) were self-treated, of which 507 (29.6%) were self-treated with antibiotics when ill. In the year prior to the survey, 2572 (23%) students reported self-medicating with antibiotics for prophylaxis.

### 3.2. Factors Associated with Keeping Antibiotics at Home

In adjusted models (Table 2), female students (aOR = 1.47, 95% CI 1.35–1.59) and those who had at least one parent with higher education (aOR = 1.27, 95% CI 1.09–1.47; aOR = 1.54, 95% CI 1.32–1.81; aOR = 1.79, 95% CI 1.51–2.13 respectively) were associated with greater odds of keeping antibiotics at home. Those having parents with a medical background were also strongly associated with a greater likelihood of antibiotic storage (aOR = 1.56, 95% CI 1.33–1.84). Students from Wuhan university were significantly less likely to keep antibiotics at home than those from Zhejiang university (aOR = 0.83, 95% CI 0.72–0.96), indicating a regional difference.

### 3.3. The Association between Keeping Antibiotics at Home and SMA When Ill

Among students who self-medicated with antibiotics when ill in the preceding month (*n* = 1711), model 1 (Table 3) shows that students from urban areas had lower odds of SMA (aOR = 0.66, 95% CI 0.49–0.88). Respondents from Lanzhou, Jilin, Nankai, and Guizhou Universities were more likely to use antibiotics for self-treatment (aOR = 2.31, 2.94, 2.29, 1.47, and 2.19 respectively, *p*-values < 0.001) compared with students from Zhejiang University. Students keeping antibiotics were about five times (95% CI 3.53–7.05) more likely to engage in SMA compared with those who did not. Model 2 shows that students having antibiotics leftover that were previously prescribed by doctors and that were purchased from pharmacies were about four times (aOR = 4.03, 95% CI 2.68–6.07) and five times (aOR = 5.29, 95% CI 3.72–7.53) more likely to engage in SMA, respectively.

### 3.4. The Association between Keeping Antibiotics at Home and Self-Medicating with Antibiotics for Prophylaxis

Among the full sample (Table 4), model 1 shows that students having parents with a medical background had 47% greater odds of self-medicating with antibiotics for prophylaxis than those whose parents did not have a medical background. A significant inverse relationship between being a medical student and self-medicating with antibiotics for prophylaxis was also found (aOR = 0.52, 95% CI 0.44–0.60). Being from an urban area was a significant predictor of having lower odds of self-medicating with antibiotics for prophylaxis (aOR = 0.80, 95% CI 0.71–0.90). Respondents from Lanzhou, Jilin, and Guizhou Universities were more likely to self-medicating with antibiotics for prophylaxis (aORs = 1.87, 1.99, and 2.18 respectively, *p*-values < 0.001). Students who had leftover antibiotics at home were 2.6 (95% CI 2.34–2.89) times more likely to self-medicating with antibiotics for prophylaxis compared with those who did not. Model 2 shows that students having antibiotics left over previously prescribed by doctors, and those having antibiotics left over previously purchased from pharmacies were more likely to SMA (aOR = 2.55, 95% CI 2.22–2.92; and aOR = 2.62, 95% CI 2.34–2.93, respectively).

## 4. Discussion

To our knowledge, this is the first nationwide study to investigate antibiotics left over at home and the association between keeping antibiotics at home and SMA in China. In addition, this is the first study to examine the association between keeping antibiotics at home and self-medicating with antibiotics for prophylaxis. In our previous study, we have identified that SMA when ill and self-medicating with antibiotics for prophylaxis contributed to antibiotic misuse as much as doctor’s prescriptions [34]. In the current study, we found that the prevalence of SMA when ill (29.6%) and self-medicating with antibiotics for prophylaxis (23%) were very high among Chinese university students, as was keeping antibiotics at home (63.1%). Keeping antibiotics at home predicted a very high chance of SMA when ill (five times) and self-medicating with antibiotics for prophylaxis (2.6 times). Based on these findings, it is crucial for China to address the massive amount of antibiotics left over at home. This may be accomplished by cutting out sources of leftover antibiotics, recycling leftover antibiotics, and educating the general public to change behaviors regarding keeping antibiotics at home.

### 4.1. Limiting Sources of Leftover Antibiotics

Regarding the sources of leftover antibiotics in China, our studies found they mainly derived from over-the-counter purchases (69.3%) and leftover medications (27.8%) from previous prescriptions.

While the high prevalence of over-the-counter purchases of antibiotics is due to a lack of knowledge in the general public regarding unnecessary antibiotic use in self-limiting disease, the easy access to over-the-counter antibiotics is also central to the situation [37,38,39,40]. Although regulations on prescriptions of antibiotics exist, they are too weak, and the implementation of such policies is generally loose in China. One of the most important cited reasons is the lack of a sufficient workforce among local authorities to monitor and supervise pharmacies [41]. Thus, street-side pharmacies usually fail to be regularly and effectively monitored for their selling practices. Driven by benefits, with no fear of light punishment [42], and sometimes under the pressure of customers, several drugstores continue selling antibiotics illegally in China [43,44,45]. With the fast development of internet and mobile technology, an electronic antibiotics sale system is urgently needed to facilitate the monitoring and supervision of illegal antibiotics sales without prescription in pharmacies. On the demand-side, which is sometimes neglected, the general public has no awareness that purchasing antibiotics at a pharmacy needs a doctor’s prescription, which puts pressures on pharmacies to violate the regulations. Greater education on this matter needs to be seen in the future. 

Although most over-the-counter purchases of antibiotics originated from antibiotic sales without prescription, antibiotics purchased with a prescription are also of concern [34]. Our findings show that 24.5% (not shown in tables) of antibiotics sold by pharmacies are prescribed by doctors in these pharmacies. Previous studies have identified that some pharmacies hired doctors solely to prescribe antibiotics to avoid being punished [44,45]. The regulation of the doctors’ antibiotic prescription behaviors in pharmacies are currently neglected or beyond the reach of health authorities. Thus, doctors hired by pharmacies should be combined into antibiotic rational prescription regulations by health authorities.

The source of previous prescriptions by doctors also plays an important role in antibiotics left over at home. In China, as is the case in other low- and middle-income countries (LMIC), antibiotics are dispensed in fixed packs instead of in an exact number of tablets, leading to massive amounts of leftover antibiotics from previous prescriptions [25]. Therefore, the antibiotic dispensing system in hospitals needs to be adjusted to replace fixed pack dispensing. Economic incentives for hospitals was a major obstacle to changing the dispensing system before the implementation of the zero-profit drug policy. This policy was implemented in community level health facilities in 2009, and subsequently has been scaled up to all public hospitals in China, making the present an ideal time to reform the antibiotic dispensing system. In addition, patients’ noncompliance with prescribed antibiotics also contributes to the problem of leftover antibiotics [29]. Our previous study has shown that over 60.5% of university students did not complete the course and stopped using antibiotics [34]. Interventions focusing on reforming the antibiotic dispensing system in hospitals and educating patients who are taking antibiotics according to the course is a pathway to address the source of leftovers from previous prescriptions.

Healthcare providers not only contribute to leftover antibiotics, but also promote the behaviors of keeping antibiotics and self-treatment with antibiotics in China. Before the health reforms of 2009, more than a quarter of institutional revenue in Chinese hospitals came from selling antibiotics [46] and these financial incentives encouraged doctors to prescribe more antibiotics, even when unnecessary, such as for treating upper respiratory tract viral infections [46]. Although subsequent health reforms have removed the profits of drug sales from the revenue of health facilities, patients have been wrongly educated to use antibiotics for self-limiting illness for over three decades. Many researchers reported that in China, as well as in other developed countries, self-medication with antibiotics was based on previous prescriptions to patients [20,21]. This led patients to expect the prescription of antibiotics during health seeking procedures and turn to non-prescription antibiotics for self-limiting diseases [47,48,49].

### 4.2. Recycling Leftover Antibiotics

Aside from cutting out the two main sources of leftover antibiotics, spreading appropriate methods for the disposal of leftover antibiotics is of the highest importance. Improper disposal of leftover antibiotics can pollute the environment [50,51]. The large quantity of leftover antibiotics should be handled in an appropriate manner. China can learn from the experiences of other countries. For example, returning unused antibiotics to the pharmacy is a common practice among Portuguese, Swedish, and Dutch residents [52,53,54]. In the United States, the Starfish Project collects unused antibiotics from households by providing free mail labels [55]. 

### 4.3. Educating the General Public to Change the Behavior of Keeping Antibiotics at Home

A campaign is needed to discourage the general public from taking leftover antibiotics at home for themselves or family members, and to encourage them to discharge leftover antibiotics responsibly. Tailored interventions can target populations at different socio-demographic characteristics, according to our findings. From our data, gender and family background were significant risk factors for keeping antibiotics at home. Female students and those having at least one parent with higher education were consistently associated with a significantly higher chance of keeping antibiotics at home, both of which support previous studies. Cortez et al. [56] and Ocan et al. [57] reported women as being more likely to keep antibiotics at home, while Awad and colleagues found that higher education levels were associated with a higher possibility of self-medication [16]. We also found that having a parent working in the medical field promoted home antibiotic storing behaviors. Usually, doctors may keep some medicine at home as a contingency for their family, thus influencing their children’s behaviors, even after leaving home. Our results also exhibit the potential mediating effect of keeping antibiotics at home and SMA. Future studies need to find whether there is a strong moderating effect between these two factors. 

There are several limitations of this study. The cross-sectional study design limited us from drawing causal inference between keeping antibiotics at home and either SMA when ill or self-medicating with antibiotics for prophylaxis. However, our study found significant associations among them and identified several important risk factors for SMA-related behaviors. Future research could further investigate a potential mediating mechanism and explore areas for intervention. Although self-reported data may contain recall bias, our findings were consistent with other research. Also, our target population consisted of university students, who are highly educated and much healthier compared to the general population; inappropriate antibiotic use among the Chinese general population could be more severe. A future study on the Chinese general population’s antibiotic use is needed. 

## 5. Conclusions

The prevalence of keeping antibiotics at home and SMA was found to be high in Chinese university students. Keeping antibiotics at home is significantly associated with SMA when ill and self-medicating with antibiotics for prophylaxis. Prescription only regulations should be strictly implemented to cut the source of leftover antibiotics from over-the-counter sources. Prescribing antibiotics by course, instead of in fixed packs, is proposed to be implemented in hospitals, as to cut the source of previous over-prescription. Attention should also be directed towards conducting tailored health education for targeted populations, aimed at discouraging them from keeping antibiotics at home.

## Figures and Tables

**Table 1:** Sample characteristics and antibiotic use behavior (*n* = 11,192).

	N (%)
**Age**	
Mean (SD)	20.7 (2.7)
**Gender**	
Male	5515 (49.3)
Female	5677 (50.7)
**Education Level**	
Undergraduate	8892 (79.4)
Graduate	2300 (20.6)
**Major**	
Non-medical	9373 (83.7)
Medical	1819 (16.3)
**Parent’s Highest Education Level**	
Illiteracy and primary	919 (8.2)
Middle school	3216 (28.7)
High school	3110 (27.8)
College and above	3947 (35.3)
**Parent with Medical Background**	
No	10214 (91.3)
Yes	978 (8.7)
**Household Income (RMB, Monthly)**	
<3000 ($461 USD)	3417 (30.5)
3000–10,000 ($462–$1538 USD)	5823 (52.0)
10,001–20,000 ($1539–$3076 USD)	1435 (12.8)
>20,000 ($3076 USD)	517 (4.6)
**Urban or Rural**	
Rural	4921 (44.0)
Urban	6271 (56.0)
**Self-Medicating with Antibiotics for Prophylaxis**	
No	8620 (77.0)
Yes	2572 (23.0)
**Keep Antibiotics at Home**	
No	4135 (36.9)
Yes	7057 (63.1)
**University**	
Zhejiang University	1775 (15.9)
Lanzhou University	1858 (16.6)
Jilin University	1961 (17.5)
Nankai University	1752 (15.7)
Wuhan University	1816 (16.2)
Guizhou University	2030 (18.1)
	*n* = 7057 ^1^
**Sources of Left-Over Antibiotics.**	
Previously prescribed by doctors	1965 (27.8)
Previously bought from a drugstore	4893 (69.3)
Given by others	125 (1.8)
Other	74 (1.0)
	*n* = 1711
**Self-Medication with Antibiotics when Ill.**	
No	1204 (70.4)
Yes	507 (29.6)

^1^ Among the full sample size, 7057 students had kept antibiotics at home. Percentages may not add up to 100% due to rounding.

**Table 2:** Factors associated with keeping antibiotics at home (*n* = 11,192).

Independent Variable	aOR (95% CI)
**Age**	1.02 (1.00, 1.04)
**Gender**	
Male	Ref
Female	1.47 (1.35, 1.59) ***
**Education Level**	
Undergraduate	Ref
Graduate	0.99 (0.87, 1.13)
**Major**	
Non-medical	Ref
Medical	1.03 (0.93, 1.15)
**Parent’s Highest Education Level**	
Illiteracy and primary	Ref
Middle school	1.27 (1.09, 1.47) **
High school	1.54 (1.32, 1.81) ***
College and above	1.79 (1.51, 2.13) ***
**Parent with Medical Background**	
No	Ref
Yes	1.56 (1.33, 1.84) ***
**Household Income**	
<3000 ($461 USD)	Ref
3000–10,000 ($462–$1538 USD)	1.15 (1.04, 1.27) **
10,001–20,000 ($1539–$3076 USD)	1.02 (0.88, 1.19)
>20,000 ($3076 USD)	1.00 (0.81, 1.23)
**Urban or Rural**	
Rural	Ref
Urban	1.50 (1.35, 1.66) ***
**University**	
Zhejiang University	Ref
Lanzhou University	1.01 (0.88, 1.17)
Jilin University	1.07 (0.93, 1.23)
Nankai University	1.11 (0.96, 1.29)
Wuhan University	0.83 (0.72, 0.96) *
Guizhou University	0.88 (0.76, 1.01)

*p* < 0.05, ** *p* < 0.01, *** *p* < 0.001. Ref: reference group; aOR: adjusted odds ratio.

**Table 3:** Factors associated with self-medication with antibiotics (SMA) when ill (*n* = 1711) ^1^.

Independent Variable	Model 1 aOR (95% CI)	Model 2
**Age**	1.03 (0.98, 1.09)	1.03 (0.98, 1.09)
**Gender**		
Male	Ref	Ref
Female	0.98 (0.78, 1.22)	0.97 (0.78, 1.21)
**Education Level**		
Undergraduate	Ref	Ref
Graduate	1.08 (0.77, 1.51)	1.06 (0.76, 1.49)
**Major**		
Non-medical	Ref	Ref
Medical	0.78 (0.58, 1.06)	0.77 (0.57, 1.04)
**Parent’s Highest Education Level**		
Illiteracy and primary	Ref	Ref
Middle school	0.82 (0.51, 1.33)	0.82 (0.50, 1.33)
High school	1.14 (0.70, 1.86)	1.14 (0.70, 1.86)
College and above	1.02 (0.61, 1.73)	1.02 (0.61, 1.73)
**Parent with Medical Background**		
No	Ref	Ref
Yes	1.01 (0.72, 1.40)	1.02 (0.73, 1.42)
**Household Income**		
<3000 ($461 USD)	Ref	
3000–10,000 ($462–$1538 USD)	0.89 (0.67, 1.19)	0.88 (066, 1.17)
10,001–20,000 ($1539–$3076 USD)	1.13 (0.75, 1.71)	1.12 (0.75, 1.70)
>20,000 ($3076 USD)	0.93 (0.53, 1.63)	0.93 (0.53, 1.65)
**Urban or Rural**		
Rural	Ref	Ref
Urban	0.65 (0.49, 0.88) **	0.66 (0.49, 0.88) **
**University**		
Zhejiang University	Ref	Ref
Lanzhou University	2.38 (1.55, 3.65) ***	2.31 (1.50, 3.56) ***
Jilin University	3.07 (1.99, 4.71) ***	2.94 (1.91, 4.53) ***
Nankai University	2.37 (1.52, 3.69) ***	2.29 (1.47, 3.58) ***
Wuhan University	1.47 (0.93, 2.34)	1.47 (0.92, 2.33)
Guizhou University	2.27 (1.44, 3.58) ***	2.19 (1.39, 3.47) **
**Keep Antibiotics at Home**		
No	Ref	-
Yes	4.99 (3.53, 7.05) ***	-
**Keep Antibiotics at Home**		
No	-	Ref
Yes (Previously prescribed by doctors)	-	4.03 (2.68, 6.07)***
Yes (Previously bought from pharmacies)	-	5.29 (3.72, 7.53)***
Yes (Other)	-	6.06 (3.06, 12.02)***

^1^ Among the full sample size, 1711 students self-treated with antibiotics in the last month. * *p* < 0.05, ** *p* < 0.01, *** *p* < 0.001.

**Table 4:** Factors associated with self-medicating with antibiotics for prophylaxis (*n* = 11,192).

Independent Variable	Model 1 aOR (95%CI)	Model 2
**Age**	1.02 (0.99, 1.04)	1.02 (0.99, 1.04)
**Gender**		
Male	Ref	Ref
Female	1.03 (0.94, 1.13)	1.03 (0.94, 1.13)
**Education Level**		
Undergraduate	Ref	Ref
Graduate	0.88 (0.76, 1.02)	0.88 (0.76, 1.02)
**Major**		
Non-medical	Ref	Ref
Medical	0.52 (0.44, 0.60) ***	0.52 (0.45, 0.59) ***
**Parent’s Highest Education Level**		
Illiteracy and primary	Ref	Ref
Middle school	0.92 (0.77, 1.09)	0.92 (0.77, 1.09)
High school	0.94 (0.78, 1.14)	0.94 (0.78, 1.13)
College and above	0.88 (0.72, 1.08)	0.88 (0.72, 1.08)
**Parent with Medical Background**		
No	Ref	
Yes	1.47 (1.26, 1.72) ***	1.47 (1.26, 1.72) ***
**Household Income**		
<3000 ($461 USD)	Ref	Ref
3000–10,000 ($462–$1538 USD)	0.88 (0.79, 0.99) *	0.88 (0.79, 0.99) *
10,001–20,000 ($1539–$3076 USD)	0.89 (0.75, 1.06)	0.90 (0.75, 107)
>20,000 ($3076 USD)	0.80 (0.62, 1.03)	0.80 (0.62, 1.03)
**Urban or Rural**		
Rural	Ref	Ref
Urban	0.80 (0.71, 0.90) ***	0.80 (0.71, 0.90) ***
**University**		
Zhejiang University	Ref	Ref
Lanzhou University	1.87 (1.58, 2.22) ***	1.87 (1.58, 2.22) ***
Jilin University	1.99 (1.69, 2.35) ***	1.98 (1.68, 2.35) ***
Nankai University	1.08 (0.90, 1.30)	1.08 (0.90, 1.30)
Wuhan University	1.13 (0.94, 1.35)	1.13 (0.94, 1.35)
Guizhou University	2.18 (1.83, 2.58) ***	2.17 (1.83, 2.58) ***
**Keep Antibiotics at Home**		
No	Ref	-
Yes	2.60 (2.34, 2.89) ***	-
**Keep Antibiotics at Home**		
No	-	Ref
Yes (Previously prescribed by doctors)	-	2.55 (2.22, 2.92) ***
Yes (Previously bought from pharmacies)	-	2.62 (2.34, 2.93) ***
Yes (Other)	-	2.72 (1.97, 3.76) ***

* *p* < 0.05, ** *p* < 0.01, *** *p* < 0.001.

## References

[1] Laxminarayan R., Duse A., Wattal C., Wattal C., Zaidi A.K., Wertheim H.F., Sumpradit N., Vlieghe E., Hara G.L., Gould I.M. (2013). Antibiotic resistance—The need for global solutions. Lancet Infect. Dis..

[2] Jacob J.T., Klein E.Y., Laxminarayan R., Cardo D. (2013). Vital signs: Carbapenem-resistant Enterobacteriaceae. Morb. Mortal. Wkly Rep. (MMWR).

[3] Tzouvelekis L.S., Markogiannakis A., Psichogiou M., Tassios P.T., Daikos G.L. (2012). Carbapenemases in Klebsiella pneumoniae and other Enterobacteriaceae: An evolving crisis of global dimensions. Clin. Microbiol. Rev..

[4] Kumarasamy K.K., Toleman M.A., Walsh T.R. (2010). Emergence of a new antibiotic resistance mechanism in India, Pakistan, and the UK: A molecular, biological, and epidemiological study. Lancet Infect. Dis..

[5] Genral Assembly of the United Nations. http://www.un.org/pga/71/event-latest/high-level-meeting-on-antimicrobial-resistance/.

[6] Inoue H., Minghui R. (2017). Antimicrobial resistance: Translating political commitment into national action. Bull. World Health Organ..

[7] World Health Organization WHO Global Strategy for Containment of Antimicrobial Resistance. http://apps.who.int/iris/bitstream/10665/66860/1/WHO_CDS_CSR_DRS_2001.2.pdf.

[8] Shorr A.F. (2007). Epidemiology of staphylococcal resistance. Clin. Infect Dis..

[9] Morgan D.J., Okeke I.N., Laxminarayan R., Perencevich E.N., Weisenberg S. (2011). Non-prescription antimicrobial use worldwide: A systematic review. Lancet Infect. Dis..

[10] Awad A., Eltayeb I., Matowe L., Thalib L. (2005). Self-medication with antibiotics and antimalarials in the community of Khartoum State, Sudan. J. Pharm. Pharm. Sci..

[11] Väänänen M.H., Pietilä K., Airaksinen M. (2006). Self-medication with antibiotics—Does it really happen in Europe?. Health Policy.

[12] World Health Organization Guidelines for the Regulatory Assessment of Medicinal Products for Use in Self-Medication. http://apps.who.int/iris/bitstream/10665/66154/1/WHO_EDM_QSM_00.1_eng.pdf.

[13] Hughes C.M., McElnay J.C., Fleming G.F. (2001). Benefits and risks of self medication. Drug Saf..

[14] Dua V., Kunin C.M., White L.V. (1994). The use of antimicrobial drugs in Nagpur, India. A window on medical care in a developing country. Soc. Sci. Med..

[15] Olayemi O.J., Olayinka B.O., Musa A.I. (2010). Evaluation of antibiotic self-medication pattern amongst undergraduate students of Ahmadu Bello University (Main Campus) Zaria. Res. J. Appl. Sci. Eng. Technol..

[16] Awad A.I., Eltayeb I.B. (2007). Self-medication practices with antibiotics and antimalarials among Sudanese undergraduate university students. Ann. Pharmacother..

[17] Scicluna E.A., Borg M.A., Gür D., Rasslan O., Taher I., Redjeb S.B., Elnassar Z., Bagatzouni D.P., Daoud Z. (2009). Self-medication with antibiotics in the ambulatory care setting within the Euro-Mediterranean region; results from the ARMed project. J. Infect. Public Health.

[18] Al Akhali K.M., Alzomar A.K., Khan N.A., Alavudeen S.S. (2013). Misuse of antibiotics and awareness of antibiotic hazard among the public and medical professionals in Thamar province, in republic of Yemen. Pharm. Glob. Int. J. Compr. Pharm..

[19] Bi P., Tong S., Parton K.A. (2000). Family self-medication and antibiotics abuse for children and juveniles in a Chinese city. Soc. Sci. Med..

[20] Lv B., Zhou Z., Xu G., Yang D., Wu L., Shen Q., Jiang M., Wang X., Zhao G., Yang S. (2014). Knowledge, attitudes and practices concerning self-medication with antibiotics among university students in western China. Trop. Med. Int. Health.

[21] Grigoryan L., Burgerhof J.G., Haaijer-Ruskamp F.M., Degener J.E., Deschepper R., Monnet D.L., Di Matteo A., Scicluna E.A., Bara A.C., Lundborgm C.S. (2007). Is self-medication with antibiotics in Europe driven by prescribed use?. J. Antimicrob. Chemother..

[22] Ocan M., Obuku E.A., Bwanga F., Akena D., Richard S., Ogwal-Okeng J., Obua C. (2015). Household antimicrobial self-medication: A systematic review and meta-analysis of the burden, risk factors and outcomes in developing countries. BMC Public Health.

[23] Okumura J., Wakai S., Umenai T. (2002). Drug utilisation and self-medication in rural communities in Vietnam. Soc. Sci. Med..

[24] Muscat M., Monnet D.L., Klemmensen T., Grigoryan L., Jensen M.H., Andersen M., Haaijer-Ruskamp F.M., SAR (2006). Patterns of antibiotic use in the community in Denmark. Scand. J. Infect. Dis..

[25] Kardas P., Pechère J.C., Hughes D.A., Cornaglia G. (2007). A global survey of antibiotic leftovers in the outpatient setting. Int. J. Antimicrob. Agents.

[26] Stratchounski L.S., Andreeva I.V., Ratchina S.A., Galkin D.V., Petrotchenkova N.A., Demin A.A., Kuzin V.B., Kusnetsova S.T., Likhatcheva R.Y., Nedogoda S.V. (2003). The inventory of antibiotics in Russian home medicine cabinets. Clin. Infect. Dis..

[27] Sawair F.A., Baqain Z.H., Abu Karaky A., Abu Eid R. (2009). Assessment of self-medication of antibiotics in a Jordanian population. Med. Princ. Pract..

[28] El Zowalaty M.E., Belkina T., Bahashwan S.A., El Zowalaty A.E., Tebbens J.D., Abdel-Salam H.A., Khalil A.I., Daghriry S.I., Gahtani M.A., Madkhaly F.M. (2016). Knowledge, awareness, and attitudes toward antibiotic use and antimicrobial resistance among Saudi population. Int. J. Clin. Pharm..

[29] Grigoryan L., Haaijer-Ruskamp F.M., Burgerhof J.G., Mechtler R., Deschepper R., Tambic-Andrasevic A., Andrajati R., Monnet D.L., Cunney R., Di Matteo A. (2006). Self-medication with antimicrobial drugs in Europe. Emerg. Infect. Dis..

[30] Zoorob R., Grigoryan L., Nash S., Trautner B.W. (2016). Nonprescription Antimicrobial Use in a Primary Care Population in the United States. Antimicrob. Agents Chemother..

[31] Togoobaatar G., Ikeda N., Ali M., Shibuya K. (2010). Survey of non-prescribed use of antibiotics for children in an urban community in Mongolia. Bull. World Health Organ..

[32] Emeka P.M., Al-Omar M., Khan T.M. (2014). Public attitude and justification to purchase antibiotics in the Eastern region Al Ahsa of Saudi Arabia. Saudi Pharm. J..

[33] Alhomoud F., Aljamea Z., Almahasnah R., Alkhalifah K., Basalelah L., Alhomoud F.K. (2017). Self-medication and self-prescription with antibiotics in the Middle East—Do they really happen? A systematic review of the prevalence, possible reasons, and outcomes. Int. J. Infect. Dis..

[34] Wang X., Peng D., Wang W., Xu Y., Zhou X., Hesketh T. (2017). Massive misuse of antibiotics by university students in all regions of China: Implications for national policy. Int. J. Antimicrob. Agents.

[35] Tian J., Hongbo S.U., Chen S., Sun X., Chen Q. (2012). Spatial-Temporal Variations of Evapotranspiration in China Mainland in Recent 20 Years. Resour. Sci..

[36] Wen Juan Xing. https://www.sojump.com/.

[37] Skliros E., Merkouris P., Papazafiropoulou A., Gikas A., Matzouranis G., Papafragos C., Tsakanikas I., Zarbala I., Vasibosis A., Stamataki P. (2010). Self-medication with antibiotics in rural population in Greece: A cross-sectional multicenter study. BMC Fam. Pract..

[38] Kamat V.R., Nichter M. (1998). Pharmacies, self-medication and pharmaceutical marketing in Bombay, India. Soc. Sci. Med..

[39] Lansang M.A., Lucas-Aquino R., Tupasi T.E., Mina V.S., Salazar L.S., Juban N., Limjoco T.T., Nisperos L.E., Kunin C.M. (1990). Purchase of antibiotics without prescription in Manila, the Philippines. Inappropriate choices and doses. J. Clin. Epidemiol..

[40] Mainous A.G., Everett C.J., Post R.E., Diaz V.A., Hueston W.J. (2009). Availability of antibiotics for purchase without a prescription on the internet. Ann. Fam. Med..

[41] Hu F., Chen M., Feng S. (2010). Generally Illegal Sale of Antibiotics among Taiyuan Pharmacy. China Qual. Miles.

[42] China Food and Drug Administration. http://www.sda.gov.cn/WS01/CL0053/24525.html.

[43] Mao W., Vu H., Xie Z., Chen W., Tang S. (2015). Systematic review on irrational use of medicines in China and Vietnam. PLoS ONE.

[44] Reynolds L., McKee M. (2011). Serve the people or close the sale? Profit-driven overuse of injections and infusions in Chin’s market-based healthcare system. Int. J. Health Plan. Manag..

[45] Sweidan M., Zhang Y., Harvey K., Yang Y., Shen X., Yao K. Rational Use of Antibiotics in China. http://www.haiasiapacific.org/wp-content/uploads/2014/03/Antibiotics-Rational-Use-report-China-2005.pdf.

[46] Xiao Y., Zhang J., Zheng B., Zhao L., Li S., Li L. (2013). Changes in Chinese policies to promote the rational use of antibiotics. PLoS Med..

[47] Trap B., Hansen E.H. (2002). Cotrimoxazole prescribing by dispensing and non-dispensing doctors: Do they differ in rationality?. Trop. Med. Int. Health.

[48] Ding L., Sun Q., Sun W., Du Y., Li Y., Bian X., He G., Bai H., Dyar O.J. (2015). Antibiotic use in rural China: A cross-sectional survey of knowledge, attitudes and self-reported practices among caregivers in Shandong province. BMC Infect. Dis..

[49] Chang J., Ye D., Lv B., Jiang M., Zhu S., Yan K., Tian Y., Fang Y. (2017). Sale of antibiotics without a prescription at community pharmacies in urban China: A multicentre cross-sectional survey. J. Antimicrob. Chemother..

[50] Young S., Juhl A., O’Mullan G.D. (2013). Antibiotic-resistant bacteria in the Hudson River Estuary linked to wet weather sewage contamination. J. Water Health.

[51] Braund R., Peake B.M., Shieffelbien L. (2009). Disposal practices for unused medications in New Zealand. Environ. Int..

[52] Ramalhinho I., Cordeiro C., Cavaco A., Cabrita J. (2014). Assessing determinants of self-medication with antibiotics among Portuguese people in the Algarve Region. Int. J. Clin. Pharm..

[53] Persson M., Sabelström E., Gunnarsson B. (2009). Handling of unused prescription drugs—Knowledge, behavior and attitude among Swedish people. Environ. Int..

[54] Blom A.T.G., De Bruijn J.C., Van de Vaart F.J. (1996). How consumers deal with the remainders of unused prescription drugs. Pharm. Weekbl..

[55] The Star Fish Project. http://www.thestarfishproject.org/?fof=Y.

[56] Cortez J., Rosário E., Pires J.E., Taborda Lopes J., Francisco M., Vlieghe E., Brito M. (2017). Antimicrobial storage and antibiotic knowledge in the community: A cross-sectional pilot study in north-western Angola. Int. J. Infect. Dis..

[57] Ocan M., Bbosa G.S., Waako P., Ogwal-Okeng J., Obua C. (2014). Factors predicting home storage of medicines in Northern Uganda. BMC Public Health.

